# Longitudinal Mechano-Sorptive Creep Behavior of Chinese Fir in Tension during Moisture Adsorption Processes

**DOI:** 10.3390/ma10080931

**Published:** 2017-08-10

**Authors:** Hui Peng, Jianxiong Lu, Jiali Jiang, Jinzhen Cao

**Affiliations:** 1 State Key Laboratory of Tree Genetics and Breeding, Research Institute of Wood Industry, Chinese Academy of Forestry, Beijing 100091, China; penghyx@126.com (H.P.); jianxiong@caf.ac.cn (J.L.); 2 College of Materials Science and Technology, Beijing Forestry University, Beijing 100083, China; caoj@bjfu.edu.cn

**Keywords:** Chinese fir, tensile creep, adsorption, swelling, unstable state

## Abstract

To provide comprehensive data on creep behaviors at relative humidity (RH) isohume conditions and find the basic characteristics of mechano-sorptive (MS) creep (MSC), the tensile creep behaviors, “viscoelastic creep (VEC)” at equilibrium moisture content and MSC during adsorption process, were performed on Chinese fir in the longitudinal direction under 20%, 40%, 60% and 80% RH (25 °C) and at 1, 1.3, and 1.6 MPa, respectively. The free swelling behavior was also measured, where the climate conditions corresponded with MSC tests. Based on the databases of free swelling, VEC, and MSC, the existence of MS effect was examined, and the application of the rheological model under the assumption of partitioned strain was investigated. The results revealed that both VEC and MSC increased with magnitude of applied stress, and the increasing RH level. Under all RH isohume conditions, the total strain of MSC was greater than that of VEC. The influence of RH level on VEC was attributed to the water plasticization effect, whereas that on MSC was presumed to be the effect of water plasticization and unstable state in the wood cell wall. In addition, the RH level promoted the relaxation behavior in MSC, while it slightly affected the relaxation behavior in VEC. In the future, the rheological model could consider the link between load configuration and the anatomic structural feature of wood.

## 1. Introduction

Creep, the study of the time-dependent strain behavior of polymeric materials, is defined as continuous deformation with time when subjected to a sustained load. In creep behavior, with the application of a given stress, an immediate elastic strain appears. Subsequently, additional long-term strain develops over time. The assessment of creep behavior (immediate and long-term strains) of polymeric materials is the primary consideration for the in-service design of polymeric materials used in load-bearing components, such as reinforced concrete floors of buildings [1,2,3]. Wood, as a sustainable building material, exhibits creep behavior because of its viscoelastic nature [4,5].

Wood, being a hygroscopic material, adsorbs or desorbs moisture with changes in environmental conditions such as relative humidity (RH) and temperature to attain equilibrium moisture content (EMC) [6,7,8,9]. The moisture movement leads to dimensional changes in wood, namely, swelling during adsorption and shrinkage during desorption. A particular characteristic of wooden load-bearing elements is that their creep behavior strongly depends on the moisture content (MC) conditions [10,11,12]. Under constant MC conditions, total deformation after a given time period is caused by normal viscoelastic creep (VEC), in which the intensity of VEC depends on constant temperature and RH conditions [13,14]. However, when wood is exposed to MC changes, it exhibits much greater deformation than wood kept under constant MC conditions, i.e., mechano-sorptive creep (MSC). The MSC was independently reported in the literature for wood in the late 1950s and early 1960s [15,16]. In general, the MSC is attributed to an unstable state in the wood cell wall when RH varies. The RH variation provides MC variation, and water molecules could form hydrogen bonds to lignin, hemicellulose, and paracrystalline cellulose of wood, resulting in the breaking and reforming of transient hydrogen bonds. Transient hydrogen bonds form free volume and localized stress in wood cell wall, which disturbs the equilibrium state of the molecular packing mode. Under an external load, the formation of free volume and localized stress accelerates the shear slip between the crystalline and amorphous phases in the cell wall [17,18,19,20].

The most evident manifestations of MSC in structural applications are increased deformation in support-bearing zones of wooden beams exposed to climate variation [21]. In wood-drying, the MSC is responsible for the relaxation of drying stresses, which otherwise might result in surface checking and cause severe losses [22]. Therefore, better knowledge of the MSC characteristics and realistic modeling of the MSC are essential to avoid undesired and unexpected deformations for the wood work components in structures.

There is common agreement that the magnitude of MSC depends on the applied stress level and increases with the cumulative MC change, or a sum of absolute values of monotonic MC changes [4]. The experimental data, although very valuable, are scattered in the literature. Still, some secondary features of MSC remain poorly understood, such as MS effect. When wood is subjected simultaneously to stress and MC changes, the MS effect may be observed as an additional deformation, i.e., MS deformation. The MS deformation as a second-order phenomenon is always accompanied by normal viscoelastic deformation and free shrinkage/swelling, and hence cannot be measured directly [23,24,25]. The free shrinkage and swelling are the result of moisture interaction within and between cell walls of wood which internally deform wood tissue. In order to quantify MS deformation, several rheological models have been proposed [22,26,27]. The general rheological model is developed under the assumption of partitioned strain, i.e., the total MSC strain is the sum of elastic strain, viscoelastic strain (time-dependent response) at constant MC, free shrinkage/swelling strain, MS strain, and thermal expansion/contraction strain [28,29]. However, the application of this rheological model is still not clearly understood because the viscoelastic properties of wood depend on many factors such as the complex anatomic structure, stress level, load model, as well as the climate conditions.

The purpose of this work is to provide comprehensive data of creep behaviors at RH isohume (RHI) conditions and under three stress levels, and find the basic characteristics of MSC. Tensile creep experiments, the VEC at constant MC and MSC during the adsorption process, should be performed in the longitudinal direction. Additionally, the free swelling will be measured, where RHI conditions correspond with MSC tests. Based on the databases of free swelling, VEC, and MSC, the existence of a secondary feature of MS effect will be examined, and the application of the rheological model under the assumption of partitioned strain for tensile creep behavior will be evaluated.

## 2. Materials and Method

### 2.1. Materials

**Figure 1:**
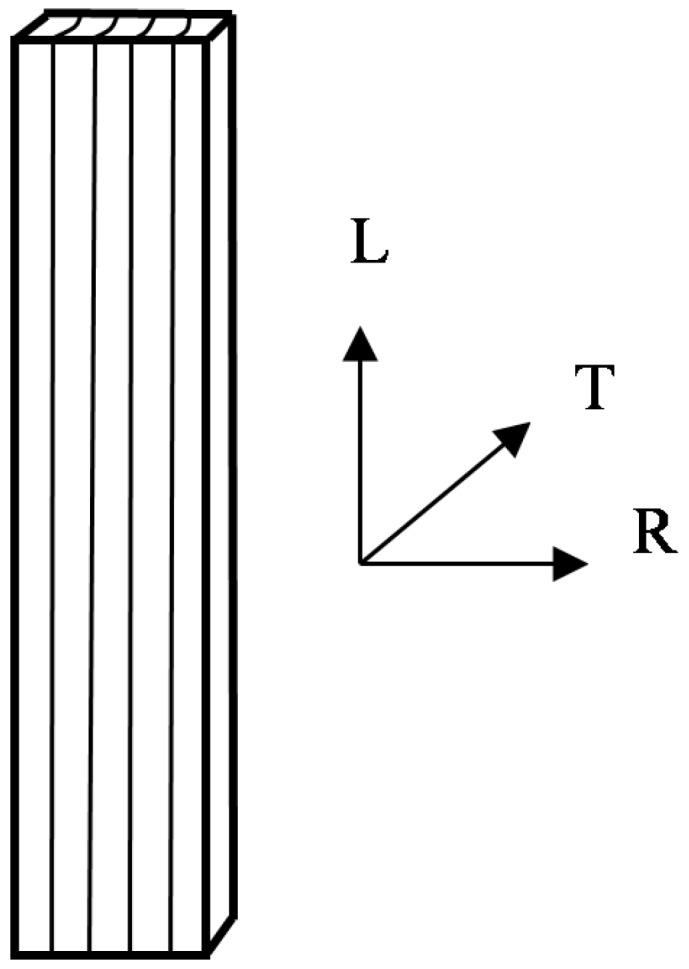
Schematic of a wood specimen.

Without any visual defects, the Chinese fir (*Cunninghamia lanceolata* [Lamb.] Hook.) heartwood specimens with a dimension of 35 (L) × 6 (R) × 1.5 (T) mm^3^ (Figure 1) were cut successively in the longitudinal direction within the 6–10 growth ring ranges. The samples were divided into 5 groups to be conditioned in a sealed container with 0%, 20%, 40%, 60%, and 80% RH at room temperature (25 °C), until constant mass was achieved. The desired 0% RH was maintained by anhydrous phosphorus pentoxide (P_2_O_5_), and the corresponding MC was about 0.6%. The other four RH conditions were attained by the sulfuric acid solution humidifying method [30]. The sulfuric acid solution with the concentrations of 57.8%, 48.0%, 36.5% and 24.1% were used to create 20%, 40%, 60% and 80% RH conditions, and the corresponding MC was about 3.5%, 6.0%, 9.3% and 14.1%, respectively.

### 2.2. Experimental Procedure

**Figure 2:**
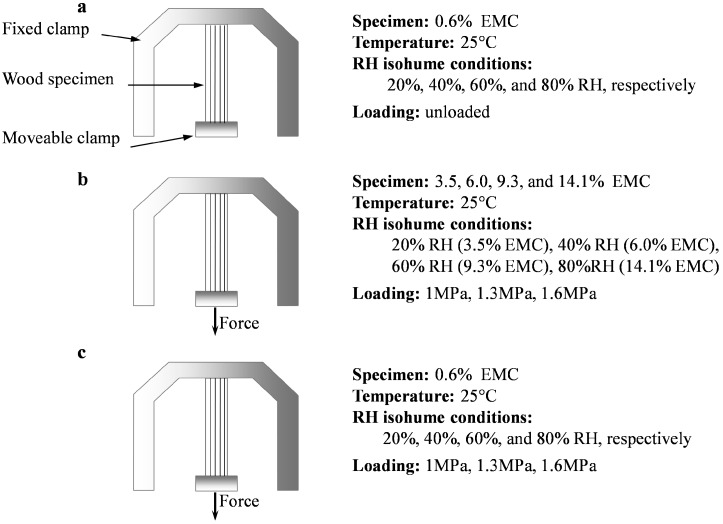
Schematic of free swelling tests (**a**); viscoelastic creep (VEC) tests (**b**); and mechano-sorptive creep (MSC) tests (**c**).

The tests were carried out by means of a dynamic mechanical analyzer (DMA Q800, TA Instruments, New Castle, DE, USA) equipped with a DMA-RH accessory. The accessory was specially designed to control the specimen environment in the range of 0 to 90% RH between 5 and 90 °C by modulating the dry nitrogen and water-saturated streams of airs in predetermined ratios. A tensile clamp with a distance of 17 mm was used. Three groups of tests on carefully matched clear wood specimens were carried out to enable separation of the principal components of longitudinal strains, as shown in Figure 2.

#### 2.2.1. Free Swelling Tests at RHI Conditions

**Figure 3:**
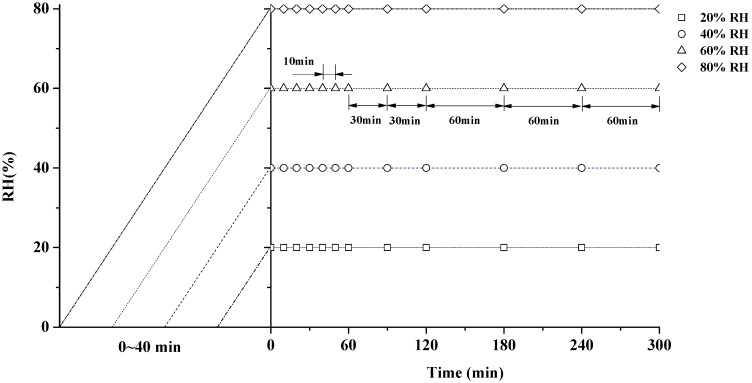
Outline of the experimental procedure at the relative humidity isohume (RHI) level of 20%, 40%, 60%, 80% RH.

The specimens with 0.6% EMC were used to measure free swelling deformation at 20%, 40%, 60% and 80% RH (25 °C). To make sure specimens were straight in tests, a pre-load force of 0.01 N was applied briefly, and hereafter, the specimens were left stress-free (Figure 2a). RH in the DMA chamber was firstly adjusted from 0% to targeted RH with the ramping rate of 2.0% RH/min. Then, the isohume conditions were kept for 300 min (Figure 3). MC was measured by weighing specimens before and after each RHI conditioning. To monitor the MC changing trend, some other specimens were also tested under the same RHI conditions, but only until the time points marked as symbols in Figure 3. Five replicates for each condition were performed.

#### 2.2.2. VEC and MSC Tests at RHI Conditions

The VEC measurements (Figure 2b) were conducted with 3.5%, 6.0%, 9.3% and 14.1% EMC at 20%, 40%, 60% and 80% RH, respectively. In addition, the specimens with 0.6% EMC were used to measure MSC at the 20%, 40%, 60% and 80% RH (25 °C). Based on stress/strain sweeps, the stress values of 1, 1.3 and 1.6 MPa were selected for VEC and MSC tests to ensure a linear viscoelastic creep behavior. In order to ensure a constant MC of the sample over the entire VEC test duration, the sample was firstly conditioned for 60 min at the matched RHI condition in the DMA chamber under a pre-load of 0.01 N to avoid buckling of the fibre. Hereafter, a load was applied for 300 min. For MSC measurements (Figure 2c), each RHI condition in the DMA chamber is also presented in Figure 3. The strain of the sample was calculated based on its elongation divided by the initial length of the sample at the beginning of the creep measurements. The total strain during a creep experiment was composed of both the elastic strain ε*_e_*, and the viscoelastic strain (time dependent strain) *ε_v_*(*t*), so that the total strain *ε_total_*(*t*), is given by;

(1) $$\varepsilon_{total}\left(t\right)=\varepsilon_{e}+\varepsilon_{v}\left(t\right),$$

## 3. Results and Discussion

### 3.1. Moisture Adsorption Behavior

**Figure 4:**
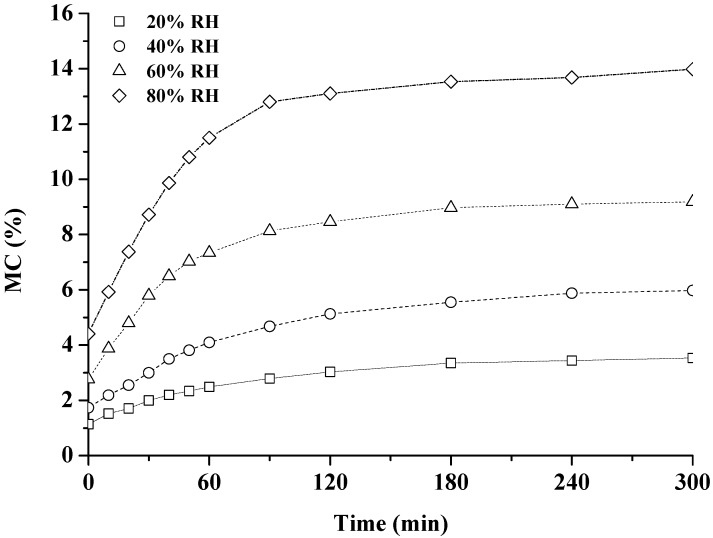
Changes of moisture content (MC) during the RHI periods (20%, 40%, 60% and 80% RH).

The time-dependent nature of wood moisture adsorption during the RHI period (20%, 40%, 60% and 80% RH) is shown in Figure 4. The increasing MC and the decreasing adsorption rate were observed for the duration of the conditioning period. The changes of MC were complete at 300 min for each RHI condition. After the RHI period of 300 min, the final values of MC were 3.5%, 6.0%, 9.3% and 14.1% at 20%, 40%, 60%, and 80% RH, respectively. Within the same isohume time, the higher the RH level, the greater the values of MC and the adsorption rate. Wood, as an open porous hygroscopic material, can adsorb atmospheric moisture due to the presence of hydroxyl groups associated with the wood cell wall macromolecules [6,31,32]. The adsorption of water in wood is the process of bound water uptake in cell walls in the local approach to equilibrium between the bound water concentration in cell walls and the water vapour concentration in adjacent macrocavities. The moisture adsorption quantity is determined by the amount of hydroxyl groups in wood, while the moisture adsorption rate is directly proportional to the concentration difference between bound water in cell walls and water vapour in adjacent macrocavities [8,9,33]. As the bound water is accumulated in the wood cell wall during the adsorption process, the concentration of bound water in cell walls increased, resulting in the decrease of the adsorption rate. In addition, the concentration of water vapour in adjacent macrocavities increased with the increasing RH level [20,34,35], confirming that a faster adsorption rate could be observed at a higher RH level.

### 3.2. Free Swelling

**Figure 5:**
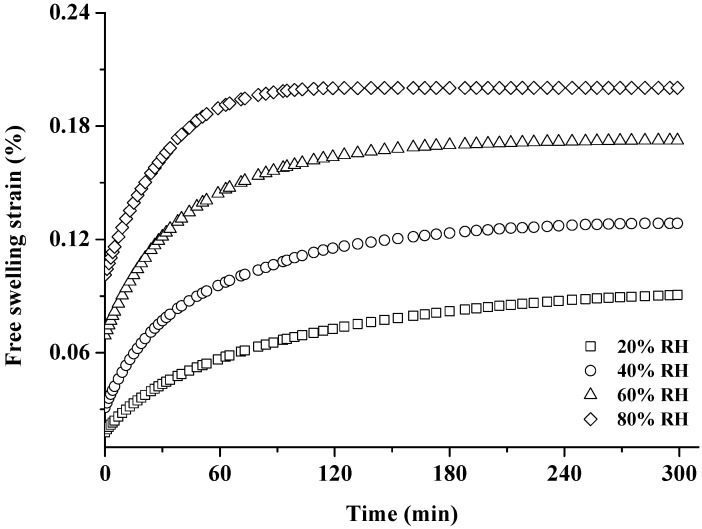
Time dependence of free swelling strain during the RHI periods (20%, 40%, 60% and 80% RH).

**Figure 6:**
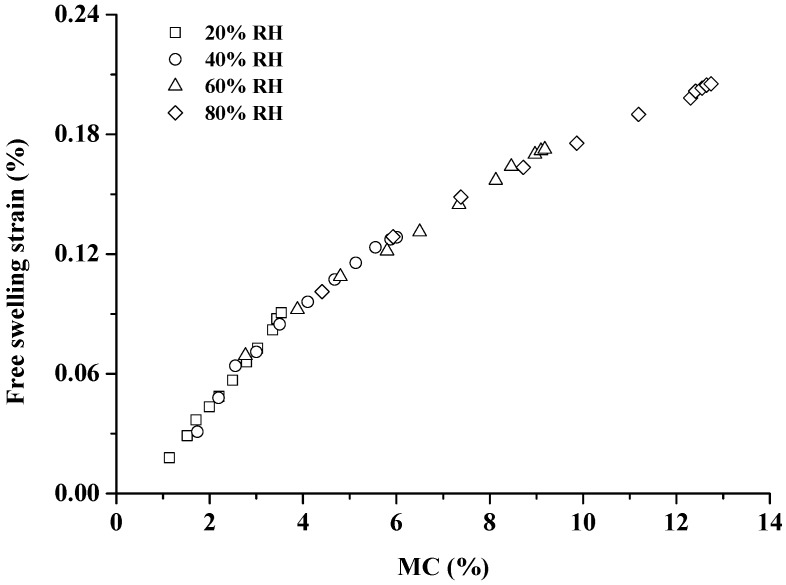
MC dependence of free swelling strain during the RHI periods (20%, 40%, 60% and 80% RH).

Figure 5 shows the free swelling strain at 20%, 40%, 60% and 80% RH. A higher free swelling strain value can be observed as a function of time. The higher the RH level, the more increment of swelling strain was found. It is evident from Figure 6 that the free swelling strain presents a positive correlation with MC, regardless of the RH level. The cell wall of wood can be viewed as a fiber composite system consisting of cellulose microfibrils embedded in the flexible matrix substances of lignin and hemicellulose. Since cellulose microfibrils possess a high crystalline content, two-thirds of the hydroxyl groups of cellulose microfibrils is inaccessible to water molecules; the abundant hydroxyl groups of the amorphous matrix are highly accessible to water molecules. Thus, the matrix dominates the free swelling during the adsorption process [36,37]. As the cell wall adsorbed moisture, the sorbed water molecules occupied the space between the microfibrils and thereby forced them apart, resulting in a change in the dimensions of wood [38,39,40,41].

A small MC gradient still possibly remained in the sample during the adsorption process, and the MC gradient would affect the free swelling and MSC due to water molecule movement inside the sample. However, from the plots “free swelling strain vs. MC” in Figure 6, the continuities between individual free swelling curves were observed. Furthermore, the thicknesses of all tensile samples used in this study were only 1.5 mm. Therefore, there can be only little MC gradient inside the sample during the adsorption process.

### 3.3. VEC at Constant MC and MSC during the Adsorption Process

**Figure 7:**
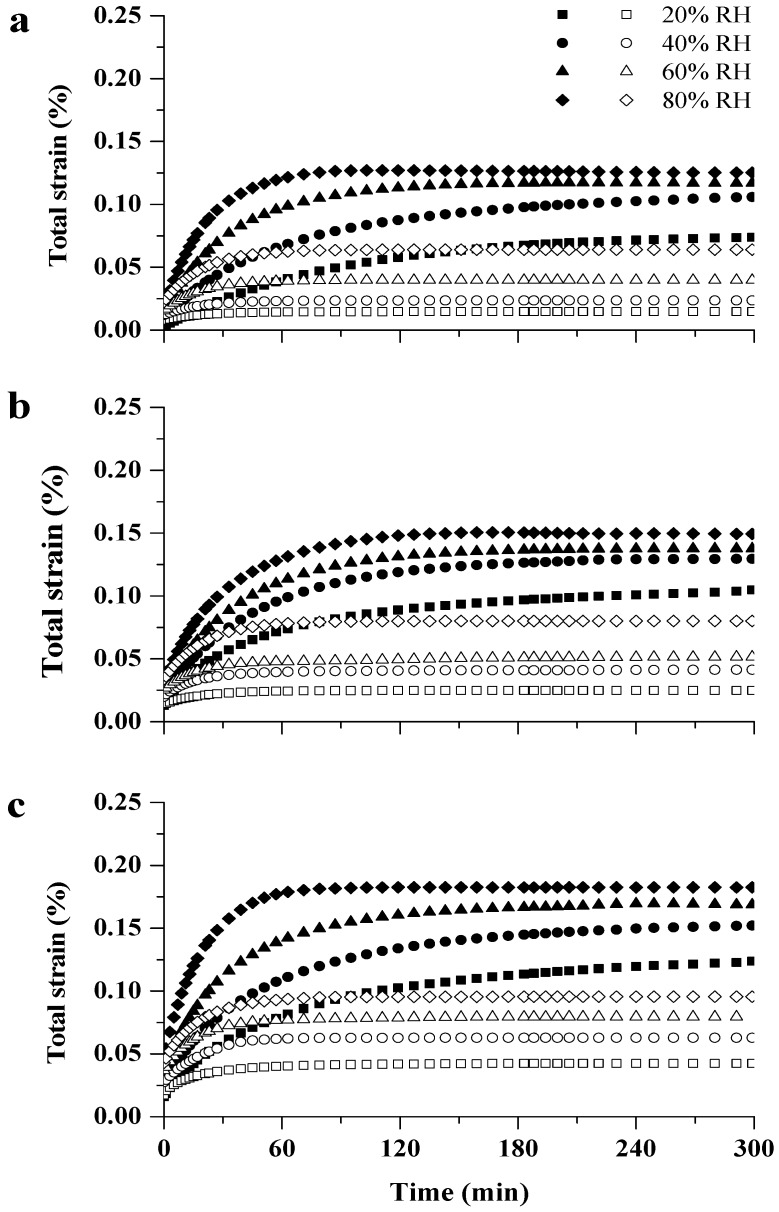
Viscoelastic creep (open symbols) and mechano-sorptive creep (solid symbols) during the RHI periods (20%, 40%, 60% and 80% RH) measured at 1 MPa (**a**); 1.3 MPa (**b**); 1.6 MPa (**c**).

Figure 7 shows the time dependence of VEC (open symbols) and MSC (solid symbols) tests at 20%, 40%, 60% and 80% RH for three stress levels (1, 1.3, and 1.6 MPa). VEC and MSC exhibited an increasing trend with time and the creep behavior highly depended on the RH level and stress level during all four RHI periods.

The values of elastic strain *ε_e_* and viscoelastic strain *ε_v_*(300) determined at 300 min creep tests for VEC and MSC under four RH levels and at three stress levels are summarized in Table 1. The higher the RH level, the greater the *ε_e_* and *ε_v_*(300) observed in either VEC or MSC. It is well known that water is a plasticizing agent for wood. With the entrance of water, some of the hydrogen bonds within the polymers matrix are replaced by bonds to water molecules, resulting in the increasing movement of the polymer network. As a result, wood at high MC is characterized by a lower rigidity than dry wood [8,9]. Creep increases caused by higher MC could be explained by the fact that water in the cell walls lubricated the slip interface. In addition, the time effect highly depended on the stress level at all four RHI periods. Increasing the loading level accelerated the creep processes; *ε_e_* and *ε_v_*(300) increased as the applied stress was raised (Table 1). The creep data under 80% RH indicated that the total strain of VEC or MSC determined at 1.6 MPa was about 1.5 or 5 times as much as that obtained at 1 MPa. The findings of these observations on the effects of time, RH level, and applied load on VEC or MSC were similar to the findings of previous research [10,14,22].

The effect of moisture on elastic behavior was different from that on viscoelastic behavior for VEC and MSC. From Table 1, it is clear that MC contributes to the difference in *ε_e_* of VEC and MSC at equal RHI level, while the *ε_v_*(300) of MSC is obviously larger than that of VEC, despite the fact that the MC of the sample is equal under all four RH levels. This may be linked to the molecular mechanisms responsible for elastic and viscoelastic responses. Whereas the elastic strain *ε_e_* is controlled by the lengthening and/or rotation of covalent and hydrogen bonds, the viscoelastic strain *ε_v_* is thought to be a result of the breaking, moving and reforming of hydrogen bonds [13,42]. Moisture within the cell wall predominately interferes with hydrogen bonds. The moisture adsorption quantity determines the lengthening and/or rotation of hydrogen bonds, while the MC changes could cause an unstable state which is associated with the breaking and reforming of transient hydrogen bonds. Hunt and Gril [17] proposed that MSC was caused by an unstable state of wood during changing processes of moisture under a stress bias resulting from moisture gradients. However, we found that the unstable state occurred during the change in MC without moisture gradients. Therefore, the unstable state of wood was caused by changes in MC regardless of the presence of stress bias. When water penetrates the cell wall, moisture molecules not only break and reform the hydrogen bonds, but also provide free volume for the rearrangement of mircofibrils and promote the “rearrangement of the hydrogen bonds” within the polymer network [18,19,20]. Samples of VEC, which had been kept under constant condition for a long time, were presumed to be more stable in the course of tensile tests as compared with samples of MSC. Each part of Figure 7 also shows that the total strain of MSC is obviously larger than that of VEC, although the *ε_e_* of MSC is lower than that of VEC. The result confirmed that the unstable state dominated the creep behavior during the adsorption process.

**Figure 8:**
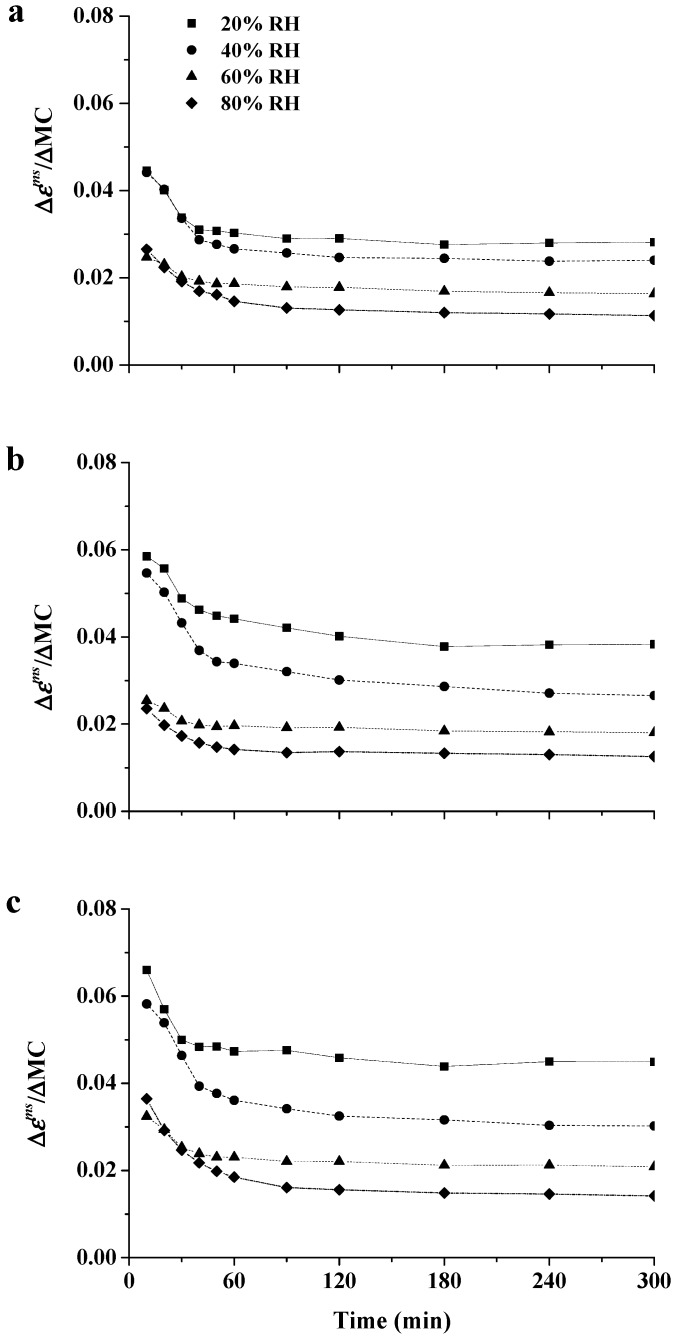
Influence of isohume time on the changes of mechano-sorptive creep per unit change in MC during the RHI periods (20%, 40%, 60% and 80% RH) measured at 1 MPa (**a**); 1.3 MPa (**b**); 1.6 MPa (**c**).

The change of MSC could be explained by the double effect of the plasticization effect of water and unstable state caused by MC changes. The parameters of Δ*ε^ms^*/ΔMC were used to evaluate the double effect and calculated as

(2) $$\Delta \varepsilon^{ms}/\Delta \mathrm{MC}=\left(\varepsilon^{ms}{}_{i}-\varepsilon^{ms}{}_{0}\right)/\left({\mathrm{MC}}_{i}-{\mathrm{MC}}_{0}\right),$$

where Δ*ε^ms^*/ΔMC is the change of the total strain of MSC per unit change in MC, *ε^ms^* is the total stain of MSC, the subscript ‘*i*’ designates the corresponding data at each RHI time point (as shown in Figure 3), and the subscript ‘0’ is for the data obtained at the beginning of the RHI period. The values of Δ*ε^ms^*/ΔMC are plotted as a function of time in Figure 8. A concave decrease of Δ*ε^ms^*/ΔMC was observed regardless of the RH level, illustrating that Δ*ε^ms^*/ΔMC decreased with the increasing MC. This result is probably attributed to two effects: (1) More rapid adsorption is associated with greater changes in creep behavior, especially early in the RHI period; (2) During the RHI period, the unstable state of the wood cell wall is mitigated and polymers can be stabilized because of the reorientation of molecular chains [43,44]. Irrespective of the stress level, the higher the RH level, the lower the value of Δ*ε^ms^*/ΔMC. The cell wall-bound water can be classified into a monomolecular water layer and a polymolecular water layer; the former is considered to be associated closely with the hydroxyl groups of wood, while the polymolecular water occurs within the transient microcapillaries and is not intimately associated with the hydroxyl groups of wood. Lenth and Kamke [45] studied the effect of these two water layers on the mechanical properties of wood, and reported that the plasticization effect on chain segments of wood polymers was more pronounced for the monomolecular water layer, and subsequently less for each additional polymolecular water layer with the increasing RH level. Figure 8 also reveals that the values of Δ*ε^ms^*/ΔMC highly depend on the stress level at all RH levels. Increasing the stress level aggravated the value of Δ*ε^ms^*/ΔMC, regardless of the RH level. This result confirmed that the positive correlation between stress and strain was not affected by moisture.

The relaxation behavior of VEC and MSC was obviously different. The relaxation time *λ*, determined by the fitting of a generalized exponential equation, was introduced to evaluate the relaxation behavior in creep tests,

(3) ε=a+bexp(−t/λ),

where *ε* is the strain of VEC or MSC; *a* and *b* are the parameters of the exponential equation. These parameters (*a*, *b* and *λ*) for VEC and MSC tests during all four RHI periods at three stress levels are listed in Table 2. In both VEC and MSC tests, stress dependency of the relaxation time was not found, regardless of the RH level. Meanwhile, the RH level affected the relaxation time of MSC but it was not associated with the relaxation time of VEC. The relaxation time of MSC exceeded that of VEC, regardless of the RH level and stress level. The relaxation time was related to the thermodynamic property of the binding energy between wood polymers and water molecules which can be quantified by the differential adsorption heat. Cao [46] measured the differential adsorption heat at equilibrium state and during the adsorption process, and stated that the slight deviation of this thermodynamic property was found at equilibrium state when MC exceeds about 4%. The average number of hydrogen bonds under non-equilibrium state was less than that under equilibrium state, and the “rearrangement of the hydrogen bonds” within the polymer network occurred during the adsorption process, resulting in a low value of the differential absorption heat, i.e., decreasing binding energy between wood polymers and water molecules and increasing relaxation time [4,11,30]. During the adsorption process, the monomolecular water layer is less ordered than the polymolecular water layer, and the binding energy between water and wood is weaker than the interaction between water molecules [30,46]. As the proportion of the polymolecular water layer increased with the elevating RH level during the adsorption process, the polymolecular water layer could be more inclined to form an additional bonding effect between themselves. Therefore, the binding energy increased and the relaxation time decreased with the increasing RH level.

### 3.4. Application of the Rheological Model under the Assumption of Partitioned Strain

Not only the swelling of wood, but also the MS strain due to moisture variation under stress was induced when moisture enters the cell wall. Strain components in wood were generally assumed as independent from each other, and hence when the effect of temperature and thermal expansion was not taken into account, the total strain of MSC (*ε^ms^*) in wood under longitudinal load was often expressed as a linear superposition:

(4) $$\varepsilon^{ms}={\varepsilon^{ms}}_{e}+{\varepsilon^{ve}}_{v}+\varepsilon^{sw}+\varepsilon_{ms},$$

where *ε^ms^_e_* is the elastic strain of MSC; *ε^ve^_v_* is the vicoelastic strain of VEC, which represents the effect of MC on viscoelastic characteristics; *ε^sw^* is the free swelling strain; and *ε_ms_* the MS strain.

**Figure 9:**
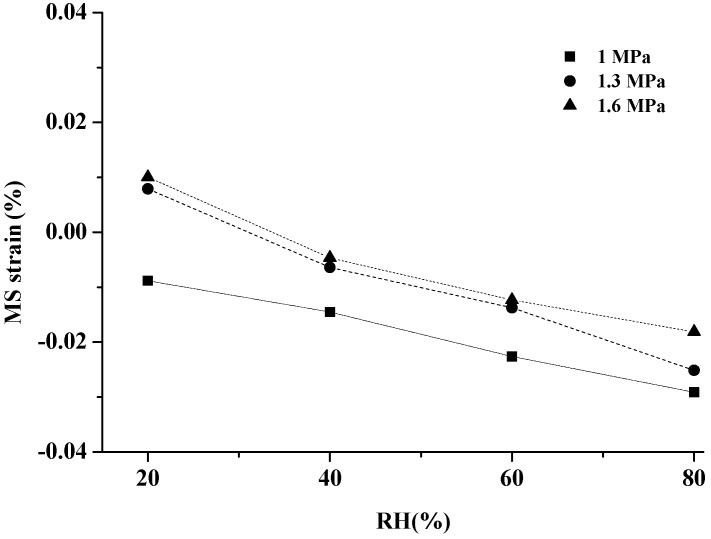
Influence of the RHI level on the MS strain (calculated by Equation (4)) at 1, 1.3, 1.6 MPa.

The resulting values of MS strain at four RH levels and under three stress levels are presented in Figure 9. The positive values evidenced the existence of MS effect, whereas the negative value was found for most specimens. On the one hand, the ultra-structural arrangement of microfibrils within the S_2_ layer of the cell wall is closely related with the MSC [47,48]. The applied load can affect the microfibril angle (MFA). When tensile stress was applied in the longitudinal direction, the external stress induced a change in cellulose microfibrils orientation and the stretching of the C-O-C bridge between two glucose molecules in a cellulose microfibril. The reorientation of cellulose microfibrils caused the cellulose microfibrils to regularly shift towards the cell axis, resulting in a decrease in the MFA [36,49]. Then, the specific interactions between cellulose microfibrils and matrix polymers in the longitudinal direction were intensified during moisture transport. The swelling in the longitudinal direction was probably restricted by the decreasing MFA because of the reorientation of cellulose microfibrils with tensile load. Therefore, the free swelling strain *ε^sw^* was higher than the swelling strain with tensile load, producing a decreasing MS strain according to Equation (4). On the other hand, the great difficulty of the aforementioned rheological model was to isolate the various effects by avoiding inopportune couplings among the anatomic structure of wood, climate conditions, and the load model [50].

Wood is described as an anisotropic natural material with unique and independent mechanical properties in three mutually perpendicular axes: longitudinal, radial, and tangential. The latter two are identified as being in the transverse direction. The arrangement of the constituents had a profound influence on the wood properties in its anatomical directions. The highly crystalline cellulose microfibrils dominated the creep behavior of the longitudinal specimen as described in this study, while the amorphous matrix was more pronounced in the transverse fibre direction. The aforementioned MFA effect could probably be mitigated in the transverse direction. These results therefore opened perspectives for future work relying on the understanding of the effect of anatomical directions on the application of the rheological model under the assumption of partitioned strain with various load models.

## 4. Conclusions

The effects of RHI level on the creep behaviors of Chinese fir were investigated at three stress levels in the adsorption phase of humidity. Furthermore, the application of the rheological model under the assumption of partitioned strain for tensile creep behaviors was examined as well. The conclusions of this study are as follows:

(1) Both the VEC and MSC increased with the magnitude of applied stress, and the increasing RH level. Under all RHI conditions, the strain of MSC during the adsorption process was greater than that of VEC at constant MC. The variation of VEC at four RH levels is attributed to the water plasticization effect, whereas the discrepancy of MSC can be seen as a result of the water plasticization effect and the unstable state in the wood cell wall. The unstable state is attributed to the formation of free volumes in the cell walls, and rearrangement of hydrogen bonds. In this study, the RH level promoted the relaxation behavior in MSC, while it slightly affected the relaxation behavior in VEC.
(2) The rheological model used to calculate the MS strain cannot be applied to the tensile creep behavior in this study. The decreased MFA under tensile stress caused the swelling under load to be lower than that in the absence of load, resulting in a smaller MS strain. More importantly, the great difficulty of the rheological model was to isolate the various effects by avoiding inopportune couplings among the anatomic structure of wood, climate conditions, and the load model.
(3) The present work added to the knowledge of creep behaviors in wood, and provided first-hand data regarding the application of the rheological model under the assumption of partitioned strain for tensile creep behaviors. These results can function, in practice, as a basis for safe structural designs of engineering structures of Chinese fir. Given the anisotropy of wood, the arrangement of the constituents had a profound influence on the wood properties in its anatomical directions. The effect of anatomical directions on the application of the rheological model under the assumption of partitioned strain must be investigated with various load models in the future.

## Figures and Tables

**Table 1:** The elastic strain *ε_e_* and viscoelastic strain *ε_v_* at 300 min [*ε_v_*(300)] for viscoelastic creep (VEC) and mechano-sorptive creep (MSC) with four RHI levels measured at 1, 1.3 and 1.6 MPa.

Parameter	Stress (MPa)	VEC				MSC			
		20% RH	40% RH	60% RH	80% RH	20% RH	40% RH	60% RH	80% RH
MC_0_		3.5%	6.0%	9.3%	14.1%	1.1%	1.7%	2.8%	4.4%
*ε_e_* (%)	1	0.007	0.012	0.016	0.022	0.002	0.004	0.012	0.017
	1.3	0.013	0.020	0.025	0.033	0.013	0.017	0.022	0.029
	1.6	0.017	0.028	0.037	0.042	0.016	0.024	0.035	0.041
MC_300_		3.5%	6.0%	9.3%	14.1%	3.5%	6.0%	9.3%	14.1%
*ε_v_*(300) (%)	1	0.008	0.012	0.024	0.042	0.072	0.102	0.105	0.108
	1.3	0.011	0.021	0.026	0.047	0.092	0.113	0.116	0.120
	1.6	0.025	0.035	0.051	0.053	0.108	0.128	0.134	0.136

Note: MC_0_, the MC of the sample at the beginning of the RHI period; MC_300_, the MC of the sample after the RHI period of 300 min.

**Table 2:** The parameters of the generalized exponential equation (Equation (3)) for viscoelastic creep (VEC) and mechano-sorptive creep (MSC) with four RHI levels measured at 1, 1.3 1.6 MPa.

Stress (MPa)	RH (%)	VEC				MSC			
		*a*	*b*	*λ*	r^2^	*a*	*b*	*λ*	r^2^
1	20	0.01	−0.01	18.50	0.99	0.08	−0.07	85.69	0.99
	40	0.02	−0.01	18.26	0.99	0.10	−0.09	58.68	0.99
	60	0.04	−0.02	17.51	0.99	0.12	−0.10	35.41	0.99
	80	0.06	−0.04	19.02	0.99	0.13	−0.11	21.35	0.99
1.3	20	0.02	−0.01	19.32	0.99	0.10	−0.08	56.45	0.99
	40	0.04	−0.02	18.33	0.99	0.13	−0.11	48.50	0.99
	60	0.05	−0.02	21.25	0.98	0.14	−0.11	39.77	0.99
	80	0.08	−0.05	19.60	0.99	0.15	−0.12	32.42	0.99
1.6	20	0.04	−0.02	20.06	0.99	0.12	−0.10	67.61	0.99
	40	0.06	−0.04	19.14	0.99	0.15	−0.12	54.80	0.99
	60	0.08	−0.04	17.58	0.99	0.17	−0.13	37.31	0.99
	80	0.10	−0.05	18.07	0.99	0.18	−0.14	19.36	0.99

Note: *a* and *b*, two constants of Equation (3); *λ*, relaxation time; r^2^, coefficient of determination.
